# Orchestrating Heterogeneous Devices and AI Services as Virtual Sensors for Secure Cloud-Based IoT Applications †

**DOI:** 10.3390/s21227509

**Published:** 2021-11-12

**Authors:** Sebastian Alberternst, Alexander Anisimov, Andre Antakli, Benjamin Duppe, Hilko Hoffmann, Michael Meiser, Muhammad Muaz, Daniel Spieldenner, Ingo Zinnikus

**Affiliations:** German Research Center for Artificial Intelligence, Saarland Informatics Campus, Stuhlsatzenhausweg 3, 66123 Saarbruecken, Germany; sebastian.alberternst@dfki.de (S.A.); alexander.anisimov@dfki.de (A.A.); andre.antakli@dfki.de (A.A.); benjamin.duppe@dfki.de (B.D.); hilko.hoffmann@dfki.de (H.H.); michael.meiser@dfki.de (M.M.); muhammad.muaz@dfki.de (M.M.); daniel.spieldenner@dfki.de (D.S.)

**Keywords:** artificial intelligence, machine learning, internet of things, cloud technology, semantic web, multi-agent systems, security

## Abstract

The concept of the cloud-to-thing continuum addresses advancements made possible by the widespread adoption of cloud, edge, and IoT resources. It opens the possibility of combining classical symbolic AI with advanced machine learning approaches in a meaningful way. In this paper, we present a thing registry and an agent-based orchestration framework, which we combine to support semantic orchestration of IoT use cases across several federated cloud environments. We use the concept of *virtual sensors* based on machine learning (ML) services as abstraction, mediating between the instance level and the semantic level. We present examples of virtual sensors based on ML models for activity recognition and describe an approach to remedy the problem of missing or scarce training data. We illustrate the approach with a use case from an assisted living scenario.

## 1. Introduction

The concept of the cloud-to-thing continuum [1] addresses advancements made possible by the widespread adoption of cloud, edge, and IoT resources. The concept involves providing digital services across multiple physical infrastructures and administrative boundaries. A key idea here is to make devices and applications at the various levels accessible in a transparent and uniform manner by providing unified access to federated and local clouds, as well as environments. To achieve this, approaches and components must be developed to explore, monitor, and orchestrate resources at various levels according to the requirements of specific use cases.

With the widespread use of IoT devices, connected and feeding large amounts of data to the internet, there is an opportunity to process these data with the most advanced artificial intelligence techniques to provide value-added services. These value-added services must likewise be integrated with existing appliances and IoT devices in a meaningful way. In addition to the challenges this poses in terms of data privacy and security, interfaces and data must also be described in a uniform manner, in order to ensure the interoperability of applications.

For the integration of heterogeneous services and IoT devices, various semantic standards and technologies have been applied to solve interoperability problems [2]. This has, on the one hand, the advantage that additional tools can be used and deployed based on the standards. On the other hand, this helps to make existing data more understandable by adding semantic markers in order to enrich and complete the data. Sensor readings are essentially incomplete, i.e., sensor data, once recorded and stored, are only meaningful and reusable with additional information about the context, e.g., time, location, and other metadata. Using semantic web technologies for this purpose is an obvious step, well supported by already available vocabularies and ontologies. The W3C semantic sensor network ontology (SSN) is worth mentioning (https://www.w3.org/TR/vocab-ssn/ (accessed on 25 October 2021)) Enriching and completing the available data are especially important, since it opens the possibility of combining classical symbolic AI and advanced machine learning (ML) approaches, where labeled data providing the ground truth is required for supervised techniques.

We consider AI services, in particular deployed ML services based on device and/or sensor data, as *second order* or *virtual sensors* providing additional information about a situation. Hence, ML services can be treated as things in the same manner as other IoT devices. Similar to virtual sensors used for, e.g., data aggregation and indirect measurement [3], virtual sensors based on ML-models constitute a suitable abstraction of (raw) sensor data and mediate between the instance level and the logical predicates and relations on the level of semantics. On the next meta-level, agent-based orchestrations combine the available sensor information to develop value-added services and applications. We therefore provide a layered approach, where the first layer gathers knowledge using physical and virtual sensors and the next-higher level complements the sensor data with semantic annotations.

In this paper, we assume local devices, physical, as well as virtual sensors employed as things, and present a unified way to make them available in federated clouds—the way up. A use case developer, however, may start with federated clouds and explore services and devices all the way down to local environments. Core components where both opposing directions converge are the thing registry and an agent-based orchestration framework, which we combine to support semantic orchestration of IoT use cases in federated cloud environments.

The paper is structured as follows: in Section 2, we describe the details of the thing registry in which we gather the information provided by the IoT devices available in the digital ecosystem and outline the security and privacy related aspects in Section 3. In Section 4, we present selected ML models and virtual sensors we developed for activity recognition in smart environments and describe in Section 5 our approach to remedy the problem of missing or scarce training data for virtual sensors. Section 6 shows how virtual sensors are made available for further usage in federated cloud applications. Section 7 presents the agent-based framework AJAN for orchestration of semantic IoT environments. We illustrate an application scenario in Section 8 with a use case from an assisted living scenario. We give an overview of related work in Section 9, discuss the results, and conclude in Section 10.

## 2. Gathering Resources in the Thing Registry

**Figure 1:**
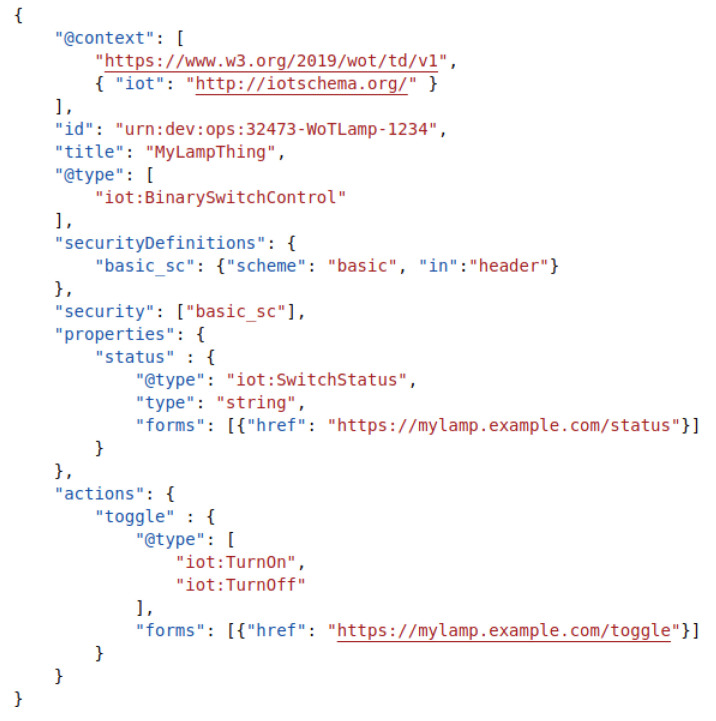
Thing description with interaction affordances.

In heterogeneous environments, IoT based data are usually fragmented. Devices and services use a multitude of different protocols and payload formats. To interconnect services and devices and enable interoperability, we use building blocks from the W3C Web of Thing (WoT) architecture (https://www.w3.org/TR/wot-architecture/ (accessed on 25 October 2021)) as well as from SENSE-WoT (For details about WoT and SENSE-WoT, in particular, we refer to [4]). The key component of the WoT architecture is the Thing Description (TD), as seen in Figure 1. The TD is a semantic description of an existing thing, be it physical or virtual, in the cloud or local. It describes metadata and interfaces of things by using a vocabulary to allow applications to integrate diverse devices.

**Figure 2:**
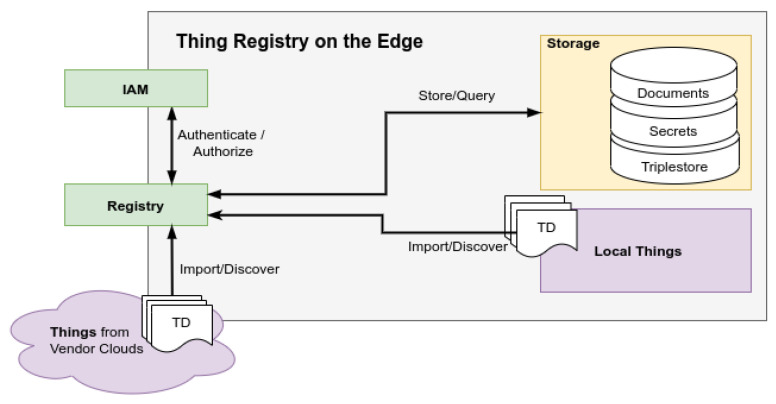
Architecture of the Registry.

The registry is responsible for discovering and generating (for legacy devices) these TDs and making them centrally available. It was developed at the German Research Center for Artificial Intelligence (DFKI) as an implementation for the WoT Thing Description Directory (TDD) (https://www.w3.org/TR/wot-discovery/ (accessed on 25 October 2021)), as seen in Figure 2. A key role of the registry is to provide multiple interfaces for querying metadata of TDs, e.g., finding all temperature sensors in the kitchen or a brightness sensor in the bathroom. The topic of user and access management plays a major key role for privacy and security, so that users are always in control over their things and data. The registry was designed to be fully integrable into existing environments, either in the cloud or on premise.

### 2.1. Discovery

By supporting the DNS-SD protocol, the thing registry can discover other services and devices. Things can load their TD into the registry after they have been discovered using CoRE Resource Directory (https://tools.ietf.org/html/draft-ietf-core-resource-directory-21 (accessed on 25 October 2021)) compatible endpoints. The registry can additionally be extended by discovery plugins. These plugins are able to discover things in the local network or cloud and generate appropriate TDs in the registry, making it possible to detect things that do not support DNS-SD and the CoRE Resource Directory endpoints. By using discovery plugins that everyone can install in their local registry instance, vendors and communities can provide an easy way to discover TDs.

### 2.2. Authentication, Authorization, and Encryption

End users of the registry must authenticate themselves via the OpenID Connect (OIDC) layer. Authorization of resources is based on the OAuth 2.0 protocol and User-Managed Access 2.0 (UMA), an OAuth based access management protocol implemented in the Identity and Access Management (IAM). We support resource permissions down to the level of affordances in a thing (actions, events, and properties). The thing registry supports internal and external encryption by using Transport Layer Security (TLS) 1.3. For a detailed view of the implemented security and privacy mechanisms, see Section 3.

### 2.3. Querying Metadata

The thing registry supports multiple storage endpoints that can be extended via plugins. This way, the registry is able to provide a wide range of different query endpoints for querying metadata of TDs. The following search methods are offered as default for the JSON-LD based information model: JSONata, JSONPath, and JSON-LD Framing.

Although these tools already allow querying metadata of TDs, SPARQL enables querying information from the TDs stored in the triple store, as TDs can be serialized to RDF. Using the SPARQL 1.1 endpoint provided by the registry allows to federate information from multiple data sources that can be mapped to RDF. Users can focus more on what they would like to know instead of how the data are structured. Additionally a reasoner is attached to the triple store to improve the quality of data integration and interoperability between different ontologies, by discovering new relationships.

### 2.4. Exposed Thing

Things are often only locally accessible, i.e., they are not available across network boundaries. Enabling remote access to these devices requires a great amount of effort and is also a security risk, as many IoT devices do not support authentication, authorization, or even encryption. Even though the TD contains all information necessary to control a thing, credentials are not part of them. In order to share a TD, credentials have to be shared too, which depending on the used security mechanism can be error prone and a security risk. To avoid these problems, an exposed thing mechanism is established. Exposed things are accessible through the registry. The registry acts as an inverse proxy to the thing described in the stored TD and injects stored credentials into the requests and, therefore, avoiding the need of sharing them manually. The sensitive data are stored in a special secret database (https://www.vaultproject.io/ (accessed on 25 October 2021)), securing, encrypting, and managing access to them. TDs of the exposed things are available in the registry. Additional steps are implemented to automatically generate homogeneous interfaces from the TDs.

#### 2.4.1. Payload Mapping

While the TDs contain semantics on how to control a thing, the returned data from the devices are most of the time heterogeneous vendor-specific JSON. In order to overcome this problem, we work on a *semantic representation* of the data, making use of the concept of *semantic interoperability* [5]. This allows us to work at a level where the syntax and structure of a transaction no longer matter, but we work on the actual “meaning” of the exchanged data. To do this, we need to *lift* data to a semantic level on which it is independent from specific implementations and structure, and, to target a specific API, *lower* it to a structured object that is consumable by such an API.

*Linked Data*, the core concept of *the semantic web* [6], already provides tools to do so, with RDF being a standard for expressing linked data in terms of triples, and URIs as identifiers for resources, it is not only ensured that each resource is uniquely identifiable, but also has a location that can be followed to, such as any standard link. As resources can also appear in the object position of a triple, we can link resources together, with the links carrying semantic meaning, spanning a *graph* that expresses the knowledge we have about that data. To lift data to a semantic level, we use a service based on *RML*. Provided with a RDF *mapping file*, structured data from XML or JSON services can be transformed into a set of triples, describing the data semantically. For lowering the data, we make use of a lowering template consisting of two parts. First, the data to be mapped are described, in terms of a graph pattern using the classes and properties defined by the ontology on the semantic layer. Second, the hierarchy of the document, as such, is defined by resources, described in terms of a slim JSON ontology. This ontology consists of classes for possible JSON data types (*object, array, number, string, boolean, null)* and the following properties:

**key:**  the key to be used for this key value pair.
**value:**  the value used for this key value pair. The value of a JSON Object can again be a JSON object or JSON array defined in the lowering template.
**data:**  if an object should refer to incoming data, this specifies the related resource of the input graph.
**root:**  the root of the hierarchy.

The registry directly implements the mapping algorithms as described above for lifting data to RDF. The RML-mapping is automatically generated using the iotschema.org ontology and semantic annotations from the TD. By using a special header when interacting with the exposed things, the lifted data are returned as JSON-LD.

#### 2.4.2. Protocol Mapping

The WoT TD ontology supports other protocols beside HTTP. These protocol descriptions are called binding templates, e.g., for COAP and MQTT. By exposing things via the registry, protocols are also mapped to a homogeneous HTTP interface, e.g., if a TD uses MQTT endpoints, these are mapped to HTTP by the registry.

## 3. Security and Privacy

Multiple security and privacy policies were developed and integrated into the architecture. When planning the requirements, a distinction was made between two main views: owners of things and application developers.

Owners of things want their things and data to e safe and protected. They also want to be fully in control of what data or things other users or applications can access, as seen in Section 3.2. For example, a thing owner wants to share the battery status of all their devices, but not the measured sensor data itself. By providing such security and privacy methods, an external service can provide the functionality to remind the owner to replace empty batteries of devices, while not being able to read sensitive sensor data itself. Additionally, the thing owner wants to share meta data from the TD by giving applications access to portions of the triple store. The battery replacement service could then query the SPARQL endpoint for additional information of the device with the empty battery, such as device name and manufacturer and, therefore, also suggest the correct battery type. On the other hand, thing owners want to share their things or specific properties, actions, or events with other users. For example, the owner of an apartment wants to make permanently installed devices, such as motion sensors and wireless light switches, available to a resident for control.

**Figure 3:**
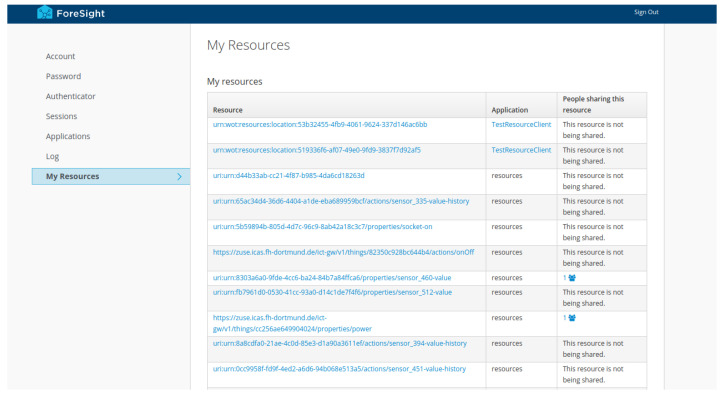
UMA 2.0 interface of Keycloak.

The application developer on the other hand wants access to control a specific device, read sensor data, or get time series data from an end user of his service. For example, an application developer can provide an appliance detection, based on smart meter data using machine learning (see Section 4.1). In order to develop such a model, the application developer needs access to training data, e.g., time series data of the smart meter. An end user of the service must then explicitly give the application access to read data from the history service, via a consent screen, as well giving explicit access to time series data from the smart meter, as seen in Figure 3. While developing a model for, e.g., predicting energy consumption, the application developer can also request access to specific resources. This way, the application developer can start building up an idea, such as building a ML model and access time series data of various sensor properties, without providing an already finished application, as seen in Section 3.3.

Well established industry protocols were used to achieve those goals. The following sections explain the aspects of the developed security and privacy policies in detail.

### 3.1. Encryption

Transport layer security (TLS) 1.3 is a cryptographic protocol that is used to securely transport data in and out the architecture by providing end-to-end encryption.

### 3.2. Authentication

All services and applications are connected to a central identity and access management (IAM) instance. In order to use applications, an end user must authenticate using the OpenID Connect (OIDC) identity layer. OIDC is part of the open standards from OpenID and allows applications to receive information and validate authenticated sessions and end users. It is an authentication layer on top of the OAuth 2.0 protocol. OAuth 2.0 is a protocol for authorization and focuses on application developer simplicity while providing specific authorization flows for web applications, desktop applications, mobile phones, and service-to-service communication. An end user of an application can use this protocol to allow an application to access their data provided by another service without revealing secrets to the client. An application can, for example, ask the end user to allow it to read their things from the registry, read their time series data, or query their metadata from the SPARQL endpoint.

Developers of applications using our architecture must register them as clients at the IAM and configure the desired data they want to access from an end user, e.g., read, delete, modify things from the registry, or read time series data. Our architecture also makes use of special service accounts that allow service to service authentication, for instance, when experimenting with data to create a new ML model. If application developers want to train a model that detects appliances based on the total energy consumption of a household, using data from the smart meter, they usually start to develop an idea using open research datasets such as REFIT [7]. Using our security and privacy approach, combined with semantic queries from the registry, application developers are able to find and request access to resources, such as smart meter data from thing owners. Thus, application developers are able to access training data from real households in early phases of research and development, before offering a finished application, by requesting permission to access a specific resource of the thing owner, as seen in Section 3.3.

The first-time end users authorize an application in our architecture via the OAuth 2.0 protocol, they have to consent to the kind of access the application wants, as seen in Figure 3. For instance, an application wants to read TDs from the registry or get specific time series data for a thing property. Consenting does not give the application full access to all resources. If an application asks for permission to read TDs from the thing registry, it cannot automatically read all things of a specific end user. The end user must explicitly authorize the application, as seen in Section 3.3.

### 3.3. Authorization

Using scopes for accessing data are just not enough, e.g., thing owners not only want to consent an application access to their TDs in the registry, but they also want to specifically authorize which TD the application can access. Thing owners want to authorize fine grained access to their owned things. They want to be fully in control of what other users and especially applications can do with their things and data. Therefore, we integrated User-Managed Access (UMA) 2.0 in our architecture to manage access rights to resources. UMA 2.0 is a OAuth 2.0 based protocol standard that is implemented in several open-source projects. The purpose of the protocol is to allow a resource owner, e.g., a thing owner, to authorize access to protected resources, e.g., things.

Authorization in our architecture works down to the level of affordances (properties, events, actions) of a thing. The owner of a thing can therefore give access to the TD itself; other users and applications are then able to read meta data from the TD. Additionally, the thing owner can restrict access to certain properties, actions, and events of a thing.

When the TD is uploaded or discovered through the registry, these resources are automatically created on the authorization server with the thing owner as the resource owner. By default, no one has access to these resources, except the owner. We use standardized REST verbs as scopes to define access to these resources: GET, POST, PUT, DELETE, and PATCH.

#### Example

As seen in Figure 1, the TD has the following resources on the authorization server:

- urn:dev:ops:32473-WoTLamp-1234
- urn:dev:ops:32473-WoTLamp-1234/properties/status
- urn:dev:ops:32473-WoTLamp-1234/actions/toggle

An application that has GET access to the urn:dev:ops:32473-WoTLamp-1234/properties/ status resource, can read its current switch status value through the exposed thing interface of the registry (see Section 2.4) as well as read the history data of it.

### 3.4. Integration

We decided to use Keycloak, an open source IAM that supports all our requirements, such as OAuth 2.0 and UMA 2.0. Besides that, Keycloak provides features, such as single-sign on, identity brokering, and user federation, as well as strong user interfaces, such as the admin console or the account management console, as seen in Figure 3, to configure UMA 2.0 access to resources.

## 4. Virtual Sensors

In this section, we present a number of implementations of virtual sensors in order to exemplify the requirements of developers for a (multi-)cloud environment. Furthermore, in Section 6, the services presented in this section will be used to illustrate the integration of services as virtual sensors into the proposed platform.

In developing a value-added service, the goal is to derive higher level information about the environment and the actors therein from lower-level sensor data. This information can be both obtained and processed at various levels of abstraction. To unify the access to and the provision information at various levels we introduce the concept of *virtual sensors*. A virtual sensor is any entity that derives information about the cyber-physical environment and feeds it back in a transmuted or augmented form. This definition encompasses both physical sensors that convert analogue information to digital signals and AI services that collect their input and publish their output in the cyber-physical environment. Each virtual sensor is part of the IoT and can be managed by the Thing Registry presented in Section 2. To illustrate the utilization of sensor information at different levels, we present a number of virtual sensors in the form of ML services from the domain of human activity recognition. All of them enrich the total power consumption (TPC) of a household in different ways.

One way to detect human activity is to derive it from the usage of certain household appliances [8,9,10], which, in turn, can be detected by the power consumption of the appliances. A reliable yet intrusive way would be to attach a measuring device to each appliance. This can be avoided with the use of supervised machine learning methods, for instance, neural networks, to disaggregate the TPC into the individual appliances. This way, the ground truth, in the form of appliance-level power consumption, is only required from a few pilot households for training and evaluation purposes.

An alternative approach by Reinhardt and Klemenjak [11] detects human activities directly from the TPC, by training load disaggregation models on human activities rather than individual appliances. In a similar manner, another route we pursue in this work is an AI service that detects the time of an inhabitant’s wake-up based on the TPC without intermediate load disaggregation. To train and evaluate the models at its base, we collect ground truth in the form of manual annotations via an IoT button from the inhabitant.

Training happens on a small number of pilot households that have agreed to share the necessary data and given the respective permissions to the developer accounts via the IAM tool. With this, the developers can browse the thing registry for eligible households and access the data via the scripting API. By listing the required permissions in the Thing Description, the developers can ensure that their service will run in households that agree to provide the data needed for its stable operation. Usually, these can be more restrictive than the ones needed for service development. In the case of those presented in this Section, the smart plugs and the IoT button are means of obtaining the ground truth for training and evaluation. In deployment, the only necessary permission is to read the live TPC data. Furthermore, if a developer wants to incorporate online-learning, he can provide either an additional version of his service with the necessary permission requirements or dynamically read from the registry what data are available about the client household.

### 4.1. Appliance Detection

To detect appliances from TPC, first, the data are acquired from a smart meter and the appliances by attaching them with the smart plugs for 45 days, from which 32 days are used for training and the rest for evaluation. We only considered the human-triggered appliances (i.e., kettle, washing machine, dishwasher, and coffee machine), as they can be used as a proxy for the identification of human activity or lack thereof.

Raw sensor data usually have a lot of problems, e.g., no constant data sampling frequency and they are prone to losses due to faulty sensors and network delays. For preprocessing, we resampled the data to have a constant frequency of 1/10 Hz. Moreover, we filled the smaller gaps of up to 2 min by interpolation and marked larger holes explicitly so the model can learn to ignore them. Once cleaned, a feature matrix χ is built by transforming TPC data into a ${\left[0,1\right]}^{NxH}$ matrix, where *N* is the number of samples and *H* is the window of history points that encodes power consumption history, after scaling them between 0 and 1. Ground truth $\Upsilon \in {\left\{0,1\right\}}^{N}$ for each appliance is formed by classifying it as either “on” or “off”. It is considered “on” if its consumption, captured using the smart plug, increases more than 1% of the maximum.

A separate binary classification model is trained individually for each appliance that learns to identify its unique power consumption signatures. Our models are inspired by seq2point [12], but have several adjustments to the input size based on the appliance type and their activity duration as detailed in Table 1. They are trained using binary cross-entropy loss and, thus, output a probability score for the appliance being in use, which is seen as a virtual sensor.

Our architecture consists of four convolutional layers and one fully connected layer. Convolution layers consist of 30, 30, 40, and 50 filters, correspondingly with stride factor 1, and a dropout rate of 0.2 is applied after each layer. The resultant vector from the last convolution layer is flattened before feeding into the fully connected layer, which consists of 256 neurons. The ReLU activation is applied to all but the output layer. The neural network parameters (i.e., layer types, number of layers, filters, etc.) along with the input window size are hyperparameters that we tuned in our experiments to achieve the best performance. These, however, are out of the scope of this study.

An appliance is considered “on” if more than 50% of the outputs in a window have higher probability score than 0.5. It can be seen in the Table 1 that the washing machine and dishwasher are easily detectable. This is because these appliances usually have longer activity duration. There are, however, more false negatives for the coffee machine, due to the particular machine having various power consumption patterns when it is in different usage stages (i.e., warming water, brewing coffee, etc.). The model trained for the kettle has the least precision since the latter is easily confused with other appliances, which results in a higher false positive rate.

### 4.2. Detecting the Time of Wake-Up

Once human-induced power consumption is detected, it is fairly certain that the inhabitant is awake. For the use-case in Section 8, however, the time of the actual wake-up is just as relevant. That is, we are interested in the length of the time interval between an inhabitant’s wake-up and their first power consumption. While this can vary, for example, between weekends and business days, we have observed this variation to be reflected in the matutinal power consumption. To abstract away the particular appliances and the constellation of their usage in the morning, i.e., order, overlap, etc., we use neural networks as a black box to estimate the above-mentioned time interval. This approach does not predict the wake-up time nor does it rely on statistical information, such as the average time of wake-up. Its main purpose is to interpret unintrusively collected live data and derive information about the situation in a smart household.

The proposed service takes care of the data acquisition via the endpoint provided by the SENSE-WoT and specified in the respective TD, does the necessary preprocessing, applies a composite model consisting of two neural networks, and cleans the output using statistical insights about the constituent models. For the training and the evaluation of the models, TPC data from the smart environment are used together with human annotations, provided via an IoT button. We chose this scenario because it allows for a relatively unobtrusive collection of ground truth. In deployment, only the power consumption data are used.

To minimize the number of tunable parameters, thereby reducing the risk of overfitting and the amount of training data required, we split the task of detecting a wake-up into two stages, each of which is covered by a separate model.

In stage 1, we use a shallow network with two 1D convolutional layers, followed by a small two-layer perceptron to classify time windows, according to whether they lie before the wake-up, whether they encapsulate a wake-up, or whether they lie thereafter. In the first and last cases, there is nothing further to be done. There, we can say that the inhabitant is either still asleep or, respectively, awake for a considerable amount of time. In the second case, we have a fixed time window, for which we can reliably say that it contains a wake-up, the time of which is to be determined. In this way, we leverage the power of convolutional layers in pattern-based classification to considerably reduce the complexity of what is, at heart, a regression problem. The classification performance is listed in Table 2. We observe a notable drop in the precision of the *asleep* class and one in the recall of the *recently awake* class. This is to be expected, since the time period, where the inhabitant is awake (as per ground truth), but does not consume energy yet, is indistinguishable from the time they are asleep.

A precise time of wake-up is then determined in stage 2 by another network consisting of one gated recurrent unit layer followed by another two-layer perceptron. It is trained independently of stage 1 on time windows that are known to contain a wake-up, since, in deployment, the other cases are covered by stage 1. Here, we leverage the ability of recurrent networks to detect temporal relationships to estimate the time gap between the time of wake-up and the first human-induced power consumption on a narrowed-down selection of energy consumption patterns. On windows that contain the wake-up time, the mean absolute error is on average 00:05:27. Since the error of stage 2 may be arbitrarily large, if applied to a window that does not contain a wake-up, it is essential to avoid this as much as possible. To this end, we select a confidence threshold of 0.97 for stage 1, below which the service does not invoke the second stage, but omits reporting a wake-up. The number is chosen this way because all of the validation samples classified to be *recently awake* with confidence 0.97 or higher were true positives; there are, sufficiently, many samples for which this is the case. Since we evaluate a new window every minute and, with a window size of 90 minutes, there is a substantial overlap between them, the main consequence of omitting a report is that the wake-up is reported at a later point in time. The window size is chosen in a way that encapsulates the time interval between wake-up and initial power consumption with a sufficiently large margin that allows the stage 1 model to make enough estimates for one of them to be above the margin. The trade-off for the upper bound on the window size is the length of the sequence fed to the GRU of the stage 2 model.

### 4.3. Summary

**Figure 4:**
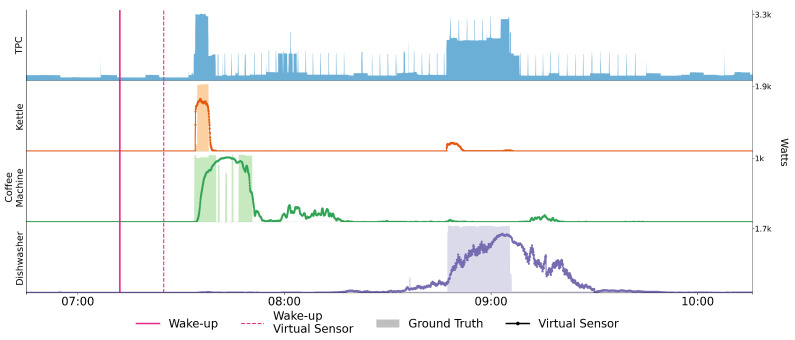
Disaggregation of TPC into different virtual sensors (scaled up for ease of visibility).

Figure 4 depicts a couple of hours in the morning where our ML services identify different appliances and the wake-up time of the inhabitant. The solid-colored filled areas show the ground truth in Watts, which is acquired using smart plugs (or smart meter in the case of TPC), whereas, line plots show the probability score for the respective device being active. Moreover, in pink, the solid and dotted vertical lines denote the wake-up time of the inhabitant from the IoT button and ML service, correspondingly. The outputs of the machine learning models, *virtual sensors*, are then deployed as separate services in the thing registry (see Section 2), and with the help of agent-based orchestration tools, such as AJAN (see Section 7), they are used in ambient-assisted living as underlying building blocks for higher level reasoning (see Section 8).

Our choice of services for the use case is, in part, due to the possibility of collecting an acceptable amount of ground truth data. This, however, is not always the case. For instance, pressing a button upon wake-up is a more justifiable effort than having to record all activities of daily living for a prolonged period of time. Therefore, a small amount of data often times has to be augmented in order to train a model on it. Augmentation techniques can vary from simple perturbations and inclusions of different kinds of noise to full on procedural generation and generative networks. Independent of the method of its generation, the registry and the concept of virtual sensors allow us to access the synthetic data in the same way we would access a real household. In the next section, we present our take on the generation of sensor data and incorporation into the proposed ecosystem.

## 5. Synthetic Sensor Data

In order to train machine learning models and make them more robust, large amounts of time series data are needed, which are time consuming and expensive to obtain in real life. For this reason, in this section, we present methods and tools that can be used to generate synthetic time series data for data augmentation based on collected real data.

### 5.1. Synthetic Power Consumption Data

As described in [13], appliances in a household can be categorized by the number of operating states.

*Constantly-on*: these include devices that always consume energy without any downtime (e.g., a WiFi router).
*Periodical*: these include devices that have periodic consumption patterns and operate independently (e.g., a fridge).
*Single pattern*: these include devices that are operated by a user and always have a very similar power consumption pattern (e.g., a kettle).
*Multi-pattern*: these include devices that are operated by a user but can have completely different power consumption patterns, depending on their programs (e.g., a dishwasher)

To serve our purpose for the different appliances, we adjust and expand this categorization. *Constantly-on* and *periodical* are still the same, but we need another categorization for appliances with more complex patterns.

One distinction we make is treating some appliances, such as a washing machine or a dishwasher, not as a power consumer, which consists of different steps, but as a process with one one power consumption pattern as output, triggered by the usage of a specific program, e.g., a short washing program. We use the possibility that a model can learn the pattern as a whole and, thus, generate different programs of an appliance. Therefore these appliances are treated as single pattern appliances, but have different classes derived from different programs, which have to be predicted. We call these *programmatic patterns*.

We make a second differentiation, since there are appliances that not only consist of different patterns, but also consume energy, as long as the inhabitant uses it. A TV, for example, is such an appliance. It starts to consume power when the resident turns it on and stops as soon as the resident turns it off. Therefore, it is not sufficient to generate a random conglomerate of different patterns. The duration of the power consumption should also be taken into consideration. We call this type *duration dependent pattern*.

There are some appliances that also consist of multiple patterns in our approach. This can have different reasons. A filter coffee machine, for example, first heats up the water, then from time-to-time releases some of the water to brew the coffee; after the coffee is made, it uses a heating plate to keep its temperature. However, after some time, it can be turned off, and the power consumption stops. This leads to very different power consumption patterns, which cannot easily be learned as one single pattern. Moreover, a fully automatic coffee machine can produce very different patterns, because it has, on the one hand, programs, such as heating or cleaning, but also user indicated process steps, such as making a coffee, for example. A stove, where the different stove tops can be used on different levels, is also an appliance with such complex patterns. These appliances produce patterns that are combined out of different patterns, such as *single*, *programmatic*, or even *duration dependent* patterns. Hence, we call these patterns *composed patterns*. This categorization is made because every category has to be handled with different techniques to generate realistic power consumption patterns. For the basic consumption, which comes from the *constantly-on patterns* and the *periodic pattern* appliances, a noisy power consumption based on the appliances in use is generated.

**Figure 5:**
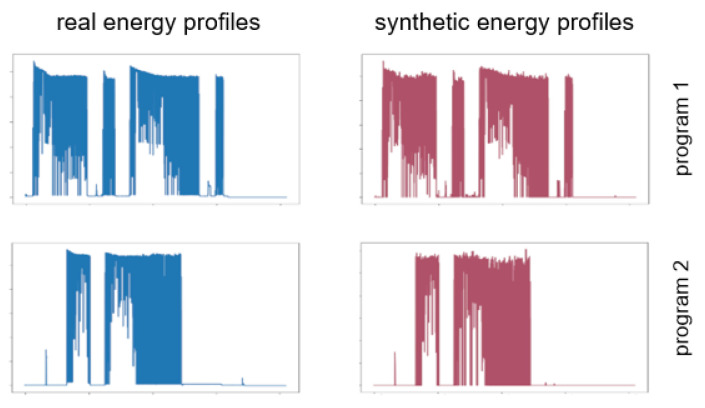
Comparison of two real energy consumption patterns from different programs of a Thermomix and two with a cWGAN-GP synthetically generated.

For *single patterns* and *programmatic patterns*, we use a generative adversarial network (GAN), to be explicit, a conditional Wasserstein GAN with gradient penalty (cWGAN-GP), comparable to [14]. The WGAN-GP framework gives us the possibility of generating realistic data based on the distribution of the training data [15,16,17]. The conditional component in the conditional WGAN-GP model (cWGAN-GP), makes it possible to not only generate random data, but also define the class that should be generated by adding labels to the input. Therefore, it is possible to generate, for example, three different programs of a dishwasher, a thermomix with multiple programs, or kettle patterns of different lengths by giving various labels as inputs for different clusters of power consumption patterns. To illustrate such a pattern, Figure 5 shows real data and synthesized data in comparison.

**Figure 6:**
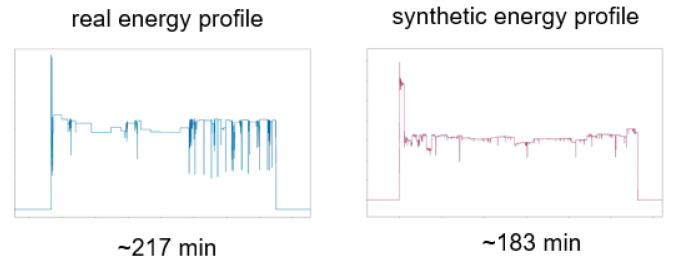
Comparison of a TV’s real (**left**) and synthetic (using XGBoost, **right**) energy consumption.

Duration dependent patterns use another model, because using a duration as a label would lead to a huge amount of labels, which could not be handled satisfactorily by a GAN. Therefore, we focus on techniques that can predict energy consumption throughout a regression. For our scenario, we use an XGBoost algorithm [18], which gets noise and duration as input and produces a power consumption pattern. This allows us to not only generate slightly different patterns with a duration that we already have in the training data but also to generate patterns that are longer or shorter than the real ones. An example of such a pattern can be seen in Figure 6, where the generated pattern has a duration, which does not exist in the training data.

To insert periodic and composed patterns, these techniques are insufficient. The consumption patterns themselves can be generated by the models described above, but to determine the starting times and the compositions of various patterns, additional information is needed. Hence, we use a sequence generation to fulfill the requirements of these categories, which is explained in the following section.

### 5.2. Activity Sequence Generation

In order to synthesize *composed patterns* (cf. Section 5.1) of devices, as well as generate more data (e.g., daily routines), we developed the *activity prediction model*. We use *long short-term memory models* (LSTM) [19], which are based on an artificial *recurrent neural network* (RNN) architecture, commonly used in speech recognition [20], exploiting the analogy between activities and sequences to words and sentences. We can use the activity prediction model to extend the data set of daily routines stemming from the existing test households, so that we get more labeled data that are as similar as possible to the existing data, but still have a certain amount of variance. This is a requirement to train ML models that can work on real data, but are also robust to data that differ slightly.

Regardless of whether we train the activity prediction model on the data of an existing or a simulated household, we use sequences of activities, where each activity is represented by a tuple, which contains the type of the activity (a domain predicate) and the time when the activity starts, measured in seconds, starting from midnight. The different types of activities, as well as their time stamps, are derived from energy consuming devices in an existing or a simulated household that can be detected by existing or simulated smart plugs (cf. Section 4). In addition, the type of activity can also be ‘wakeUp’ or ‘fallAsleep’, provided that a device (e.g., a dedicated button activated by an inhabitant) in the test household can be used to track when the resident wakes up or goes to sleep.

In addition to generating daily routines, the activity prediction model can also be used to generate the *composed patterns* (cf. Section 5.1) of devices. The patterns of a fully automatic coffee machine (e.g., heating phase, brewing process, rinsing process) for example, are nothing other than temporal sequences of activities that can be generated with the activity prediction model on the basis of recorded real data. Furthermore, the activity prediction model can be used to generate the cycles of an device with a *periodic pattern*, for example the cooling cycles of a fridge.

**Figure 7:**
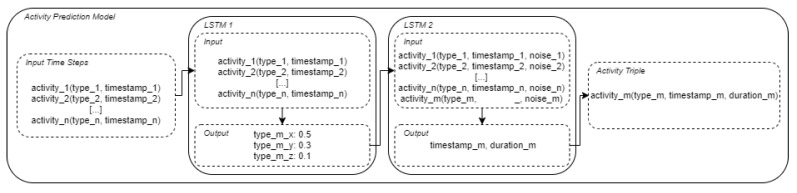
Activity Prediction Model Example.

Let us now consider the example depicted in Figure 7, where we generate a new sequence of activity triples. To do this, we iteratively generate one activity triple after the other with the activity prediction model. The activity prediction model considers the last *n* activity tuples (activity_i(type_i, timestamp_i) with n = [1, 2, …, n] (cf. *Input Time Steps*)) to generate the next activity triple.

The activity prediction model consists of two LSTM networks, where the first LSTM (*LSTM 1*) predicts the probability of the next activity type based on the types of the last *n* activities of a sequence, similar to a simple *ngram* approach [21]. An *ngram* model is a probabilistic approach, often used in computational linguistics, which calculates the probability of the next word based on the last *n* words by counting occurrences in the training data set. The advantage of our LSTM-based approach is that we use not only the last *n* activity types for prediction, but also the corresponding time stamps (cf. Figure 7, *LSTM 1—Input*). Depending on the previous activities, *LSTM 1* generates a probability distribution for the occurrence of the activity type as an output based on which we then select the type of the next activity randomly according to the distribution, resulting in activity sequences that display some variance. Depending on the dropout and the number of episodes we train *LSTM 1* with, we can create more or less variance. In our example in Figure 7, we get a 0.5 probability for *type_x* and only a 0.3 probability for *type_m_y* as an output of *LSTM 1*, but *type_m_y* is chosen for our next activity.

To generate the time stamp and the duration we use another LSTM network (cf. *LSTM 2* in Figure 7), which gets the same input as *LSTM 1* as a basis, in addition a new input triple with the predicted activity type as a time step (activity_m(type_m, _, noise_m), where *type_m = type_m_y*). We use the predicted activity type from *LSTM 1* as an additional input for *LSTM 2*. In order to predict the time when the activity starts, and its duration, *LSTM 2* needs the knowledge of what type the activity is. Since we generate the time stamp and the duration of *activity_m* in this step, a placeholder is passed instead. To generate more variance, we add a noise parameter to every time step. In our example, *LSTM 2* generates *timestamp_m* and *duration_m* as output.

With this, an activity triple (*activity_m(type_m, timestamp_m, duration_m)*) is generated and appended to the already generated sequence. If we generate the next activity with our activity prediction model as described above, the triple just created is also used for this purpose. With this approach, we can quickly and iteratively build representations of daily routines that we can then use further to generate the labeled power consumption of a household. Such a sequence of activities resulting in a daily routine can, for example, appear as follows:

[’wakeUp’, 15780, 1]
[’kettle’, 17520, 185]
[’coffeeMaking’, 17700, 30]
[’kettle’, 40860, 132]
[’tvWatching’, 66240, 7153]

### 5.3. Synthetic Labeled Time Series Data

To combine the generated action sequences (cf. Section 5.2) and the generated power consumption data (cf. Section 5.1) to create labeled time series data, which then can be used to train machine learning services, we use the tool *SynTiSeD* (Synthetic Time Series Data Generator).

*SynTiSeD* is a simulation tool for generating smart home data that was developed at DFKI using Unity 3D (https://unity.com/ (accessed on 25 October 2021)). With this tool, a user can first load a 3D model of an apartment or the floor plan as an image, which is then converted into a 3D model, through a graphical user interface (GUI). After that, appliances can be placed in the apartment, such as a kettle, coffee maker, dishwasher, etc. In addition, with a menu, the user can specify whether the apartment should have passive consumers (mainly appliances of the categories *constantly-on* and *periodical*, such as a fridge, router, warm water pump, etc.), which are in use for the duration of the simulation. Each smart plug (represented by the models described in Section 5.1) placed in the apartment, as well as the smart meter (the aggregated output of these models), are described with a Thing Description, which is then managed by the thing registry.

Once the user added a resident to the apartment, action sequences can be assigned to the resident to be performed in the apartment. These action sequences can be derived from the activity prediction model described earlier (cf. Section 5.2), but the user can also assign actions to the resident manually using the GUI.

The labeled time series data can be created by simulating the resident in the apartment performing the previously assigned actions. Whenever the resident performs a kettle activity in the simulation, the corresponding kettle model described in Section 5.1 and located in the registry is addressed dynamically. The model then provides a simulated kettle power consumption, which is then fed into the smart meter of the apartment. The same applies to all power consumers (both active and passive) used by the resident during the simulation in the apartment. The results are labeled time series data that are exported by *SynTiSeD*, e.g., every second, containing the generated smart meter data and all devices used in the simulation, as well as the activities the resident performed, which serves as ground truth to enable supervised learning.

Because all sensors (smart meter and smart plugs) of the apartment can be described with Thing Descriptions managed by the registry, and all data can be generated by *SynTiSeD* in real time, the simulation can be used as a full substitute to a real household and, therefore, be used to train and test machine learning models or other applications. This can be particularly helpful, if there are not enough data from an individual household, which is often the case in practice. The models used for power consumption generation in such a simulation do not have to be trained every time an inhabitant changes appliances or moves in a new flat. It is possible to register models that can generate the power consumption patterns of a specific appliance model of a manufacturer (cf. Section 5.1). Therefore, one can browse for these ready-to-use models, which have the possibility of generating the power consumption of individual households for a simulation. This simulation is then based on all appliances it uses, without having real smart plugs installed for the fine-tuning and testing phase of ML models.

**Figure 8:**
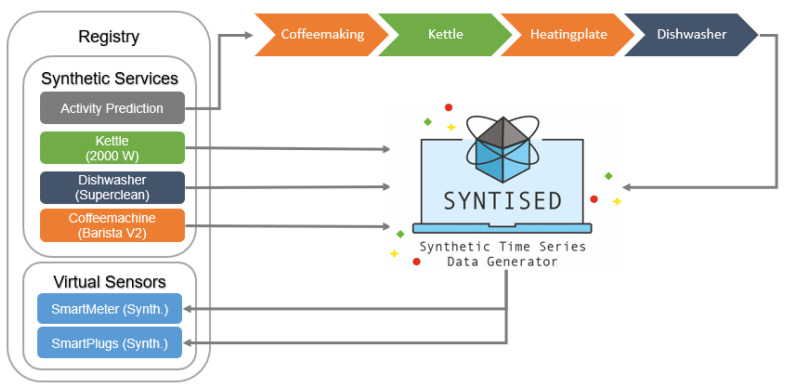
Architecture of synthetic generation of power consumption data with help of *SynTiSeD*, including services from the registry.

Figure 8 shows the architecture between the registry and *SynTiSeD*. In this example, the power consumption generation models (cf. Section 5.1) of three devices have been registered in the registry, namely a kettle (2000 W), a dishwasher (Superclean), and a coffee machine (Barista V2). Furthermore, the activity prediction model described in Section 5.2 was also registered. At the request of *SynTiSeD*, the activity prediction model now provides a sequence of actions for a resident to be simulated in an apartment. During the simulation, the simulated resident executes the activity sequence. If they use power consuming devices during this time, *SynTiSeD* automatically requests the power consumption models for these devices, which are stored in the registry and receives power consumption patterns from them, which then combines in the smart meter. All devices used by the resident during the simulation (simulated SmartPlugs) as well as the smart meter (simulated SmartMeter) are described with a Thing Description, and registered in the registry as things of a household. Thus, at any time during the simulation, used sensors can be queried via the registry, such as the ones from a real household.

## 6. Integrating Virtual Sensors into the Registry

Using our proposed architecture, we developed multiple ML models for sensor-based activity recognition (cf. Section 4), as well as models for simulated sensors and activities (cf. Section 5). In order to use them in applications, we must encapsulate them into services, e.g., HTTP microservices. These web services are very lightweight and simple to containerize and, therefore, easily deployable into the most common container–orchestration systems, such as Kubernetes, OpenShift, or Docker Compose. Another benefit of using microservices is the scalability. Not only can microservices handle a large number of requests, but they are also prepared to handle even more in the future through horizontal scaling, if necessary. Therefore, all our ML models are encapsulated into containerized microservices as virtual sensors, so that instances can be created as needed, e.g., per building, household, or even per user.

The developed models all require specific input to classify or predict events, e.g., the appliance detection, as well as the wake-up time detection model (cf. Section 4), need the energy consumption measured from a smart meter as input. The synthetic data services, on the other hand, need input, such as the duration of a specific activity (cf. Section 5). Some input can be optional, e.g., the program of an appliance, which should be synthesized, can be selected manually or picked randomly by the service itself. Input is handled through HTTP resources of the microservices by using the body of POST and PUT requests. As the payload format, we mostly use JSON, or, where feasible, directly use RDF based formats, such as N-Triples, N-Quads, or JSON-LD.

We use TDs to semantically describe virtual sensors the same way as we describe physical sensors. In this section, we demonstrate how to use TDs to describe input and output of actions, capabilities, and other metadata to easily find and use the encapsulated ML models.

The W3C Thing Description Template (TDT) is a description for a class of things, and used to describe the common metadata and affordances of virtual sensors. These templates look much like the TD shown in Figure 1, except that they lack the form elements to the final resources of each virtual sensor instance. By combining this template mechanism with package managers, such as Helm (https://helm.sh/ (accessed on 25 October 2021)), we are able to automatically spawn instances of these virtual sensors for users, as well as automatically register them at the thing registry.

**Figure 9:**
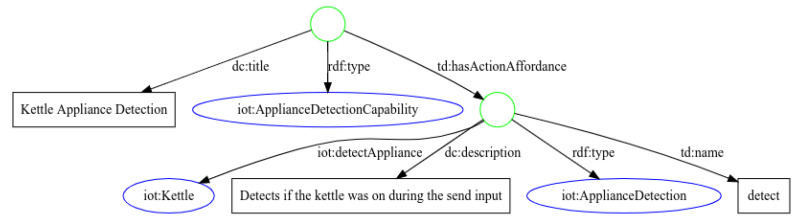
Excerpt of a Thing Description template for the kettle appliance detection service.

As an example, we provide the TDT for the service encapsulating the kettle appliance detection model from Section 4.1. As shown in Figure 9, the template describes a thing with a capability to detect appliances, by extending the *iotschema.org* capabilities with a new class iot:ApplianceDetectionCapability. The *detect* action from the TDT is using a new action class iot:ApplianceDetection; this indicates that the following action is able to detect appliances. The exact detectable appliance is described using the iot:detectableAppliance property, in this case IoT:Kettle, which is a subclass of IoT:Equipment.

The input of the *detect* action is defined with the default *iotschema.org* and *schema.org* vocabularies. iot:ActivePowerData and schema:DateTime indicate that the microservice is expecting JSON input in the form of an array, with active power data, with creation timestamps tuples.

The output of the *detect* action defines a new sub class iot:ProbabilityData of schema:PropertyValue representing a probability value between 0 and 1. As described, the service outputs an array of probabilities with the corresponding timestamps, indicating the probability that the kettle was running at that moment in time.

If the virtual sensor is created, the forms and security definitions are automatically added to the TDT and point to the newly instantiated microservice. The resulting TD is then added to the thing registry. From this point, application developers, in compliance with the security and privacy mechanism (cf. Section 3), are enabled to find the virtual sensor, using, e.g., the SPARQL endpoint of the thing registry. Additionally, proposed tools, such as payload mapping and lifting (cf. Section 2.4.1), and the exposed thing mechanism from Section 2.4, make it possible to seamlessly integrate virtual sensors into our multi-agent system AJAN, as presented in the next section.

## 7. Orchestrating Things and Services with AJAN

The multi-agent system (MAS) paradigm is well suited to implement a higher value “intelligent” functionality of semantically described heterogeneous domains on an application level, while hiding the deployment context from the user. It has already proven that it can be used to realize advanced distributed applications in environments with a high diversity, such as the WoT, see [22,23,24]. With the MAS framework, AJAN, we present a modular web service with which autonomous LD based system behavior can be modeled and executed.

Accessible Java Agent Nucleus (AJAN, see [25]) (https://github.com/aantakli/AJAN-service (accessed on 25 October 2021)) developed at the German Research Center for Artificial Intelligence (DFKI) is a MAS to manage knowledge-based agents. AJAN was primarily developed to interact in LD environments; thus, AJAN uses RDF triplestores to store agent knowledge and SPARQL to query it. The entire AJAN agent model is defined in RDF, as is the agent behavior. The agent behavior model used in AJAN is SPARQL-extended behavior trees, called SPARQL-BTs. With these SPARQL BTs, an AJAN agent has the ability to dynamically explore the agent domain and query and orchestrate LD-based domain resources. With the AJAN-editor (https://github.com/aantakli/AJAN-editor (accessed on 25 October 2021)), AJAN provides a user-friendly graphical user interface (GUI) for modeling agents and their behaviors. Thus, this editor can be used to define the orchestration of LD resources at the design time and can be used at runtime to create and execute agents.

### 7.1. AJAN Agent Model

**Figure 10:**
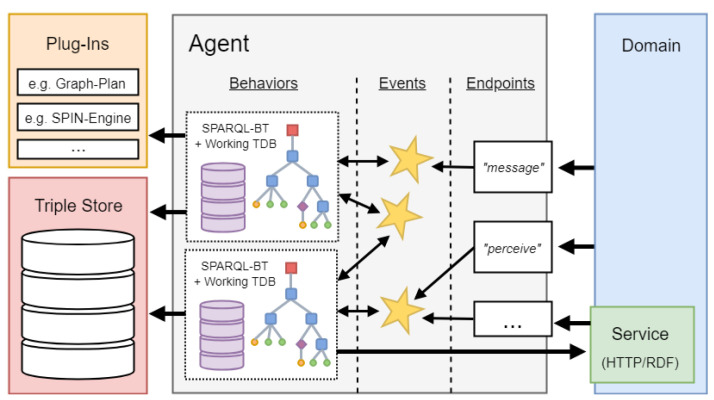
Schematic structure of the agent model [25].

An AJAN agent (see Figure 10) has one or more behaviors, each executed in a single thread and consisting of a SPARQL-BT (see Section 7.2) and a corresponding RDF based execution knowledge (EK), which stores internal behavior knowledge (e.g., procedural variables); one agent specific RDF based knowledge base (KB), storing internal agent knowledge (e.g., the agent status), which can be accessed by all agent behaviors (unlike EKs, which can only be accessed by the respective SPARQL-BT); one or more events, each holding RDF data in the form of named graphs; and one or more agent endpoints. These endpoints are the agent interfaces to its domain and forward incoming RDF messages as events. Behaviors are linked to such events. If an event occurs, the behaviors linked to it are executed. While executing a SPARQL-BT, it can access special incoming event data by querying its named graph. Each behavior can also create events to trigger other behaviors. A detailed description of how to create an AJAN agent model can be found in the AJAN Wiki (https://github.com/aantakli/AJAN-service/wiki/AJAN-Agent-Template (accessed on 25 October 2021)).

### 7.2. Behavior Trees for Orchestration

For modeling agent behavior, AJAN uses the SPARQL-BT (SBT in short) approach, an extension of the well known behavior tree (BT) paradigm with SPARQL queries. The BT approach is designed to define agent behavior or its decision-making process. BTs are characterized by their modular and graphical properties, making them highly extendable and easy to use. This paradigm with loops, sequences, parallels, and an implicit knowledge base is often described as a combination of decision trees with state machines [26]. Due to space limitations, we refer the reader to [27] for a detailed overview of the behavior tree paradigm. Basically, BTs are used in AJAN to perform contextual SPARQL queries for state checking, updating, constructing RDF data used for action executions, or to control the internal execution of an AJAN agent behavior. SBTs are defined in RDF, whereby a semantic description of the behaviors they implement is available. SBTs use standard BT composite and decorator nodes and are processed as typical BTs (https://github.com/libgdx/gdx-ai/wiki/Behavior-Trees (accessed on 25 October 2021)), with the difference that SBTs are executed via events. How to model SBTs, as well as which SBT nodes are available, can be found in the AJAN Wiki (https://github.com/aantakli/AJAN-service/wiki/SPARQL-Behavior-Tree (accessed on 25 October 2021)).

### 7.3. Incorporating Things

**Figure 11:**
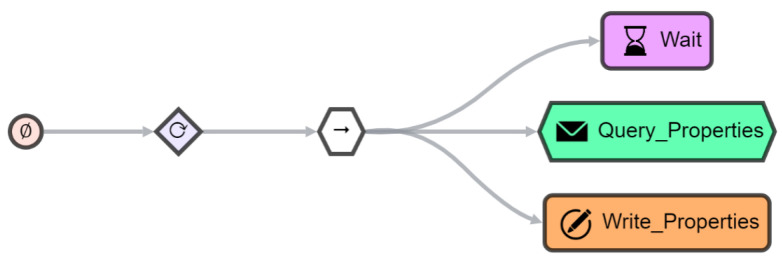
SBT of a *monitor agent*.

The registry acts as the connector between the TDs and AJAN. Once a TD is available for a device or service in the registry, a *monitor agent* is initialized in the AJAN runtime environment. Such an agent periodically queries the endpoints defined in the corresponding TD and writes the received real-time thing properties into the registry as RDF graphs. The SBT of a *monitor agent* (see Figure 11) consists of a *sequence node* (sequences are visualized by an arrow icon) of three leaf nodes that are executed by a *repeater node* iteratively. The first leaf node is a *wait node*, waiting as long as the query time described in the related TD. After that, a *query node* executes an HTTP GET request to the endpoint described in the TD and converts the returning JSON into an RDF format. As described in Section 2.4, this lifting is realized using RML. Finally, the *write node* stores the value received as RDF in the appropriate position in the TD located in the registry.

In this way, data or global domain knowledge is built up with the registry, containing all TDs, as well as the queried values. The domain knowledge is present in the form of a triplestore. On this knowledge, specialized AJAN agents, called *functional agents*, can again be initialized to work with this knowledge. Unlike a *monitor agent*, a *functional agent* is not limited to reading data. The behaviors of such an agent are much more complex. For example, they use the information provided by a *monitor agent* for decision-making and actively controlling the agent’s domain.

## 8. Use Case

As a use case, let us look at an example in the area of assisted living. A developer wants to design an application that notifies caregivers in the event of a resident’s anomalous inactivity, and gives the former access to the apartment in the case of a confirmed emergency. The necessary devices and services are distributed on several clouds, for example, a door lock and face recognition service in the cloud of the responsible housing association, while the resident’s device services run in the cloud of a device provider, or in the local network. The developer uses semantics on multiple different levels, in order to implement such a use case in our scenario. In a first step, the resident has to authenticate against the IAM instance and give the application the appropriate permissions to query metadata from the devices and operate them. In this specific case, the developer needs access to the energy consumption data measured by smart plugs in the resident’s apartment as well as a smart lock. By using the metadata from the TDs and the SENSE-WoT location service, the developer can use the semantics to query which sensors are available.

**Figure 12:**
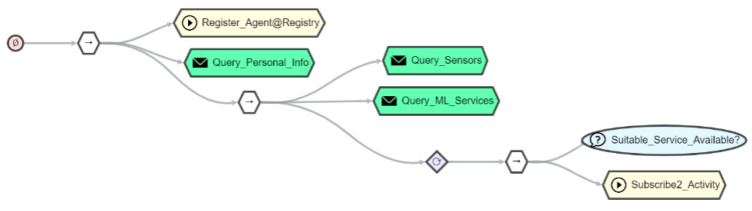
SBT for subscribing to ML-services and registration at the registry.

To interconnect these things with other services, the developer uses AJAN as a visual programming tool that uses the semantics on how to operate and transform heterogeneous vendor specific payloads of these things. To detect an emergency with AJAN, an advanced *functional agent* can be modeled that uses the registry and multiple services, across multiple clouds, to listen for activity messages and to react to them. For this purpose, the authenticated resident needs to register AJAN as a trusted client. The resident may decide to acknowledge and accept giving AJAN access to specific resources protected by scopes, e.g., reading thing metadata or use the exposed thing interface to receive lifted payloads. After authentication, see Figure 12, the agent can use the identity information received, in form of an access token, to query the registry (*Action Node*) and read out the resident’s personal data (first *query node*), encoded in the token, to enable direct communication with the resident. Afterwards, TDs are queried from the registry so that the agent can decide which activities could be recognized with existing sensors. Next, the agent searches for the required services in the registry, e.g., one trained for a specific coffee machine model of a manufacturer, and inform a service from which sensor address it can get the required input data. After this registration, the agent is ready to receive the events from the service.

Five pre-trained ML-services are employed in our use case; four of them classify human activity via household appliances and the other detects the wake-up time, as presented in Section 4. As these services are pre-trained and might not work on every appliance model of any manufacturer, but also since the daily routines of individual residents are very different, it is beneficial to fine-tune the models on the data from the flat they are used in. However, not every flat has smart plugs installed or a button to determine the wake-up time. Hence, there may be no possibility to enrich the smart meter data with ground truth. Even if these devices were installed, there would be no certainty that there would be enough data available to train ML-Services. Therefore, *SynTiSeD* (see Section 5.3) can be used to generate power consumption data (as a fallback, if there is no ground truth available, the inhabitant may provide an overview of daily routines, which can then be augmented by the activity prediction model) with help from the services mentioned in Section 5.1 and Section 5.2. The activity prediction model can directly generate sequences based on the historical data and augment the existing data set. These sequences can then be used as ground truth and to synthesize the total power consumption. This can be done by selecting appropriate models for appliances used in the flat, from the registry (see Section 2), which can generate power consumption patterns for the specific activities. Based on these data, the classification service as well as the wake-up detection service can then be fine-tuned and are ready to use. The classification ML-services use time series of power consumption data of a smart meter to detect the activities of a device. Every time an activity occurs on these appliances, the classification service pushes these classified events, lifted by our mapping approach (see Section 2.4), as RDF messages to the agent, enriched with semantic information of starting and ending time and the activity type. The wake-up time service uses data from the smart meter to specify the time an inhabitant wakes up, and sends this information to the agent as well. Based on these data, the agent possesses knowledge about human activities in the environment, and can use it to detect unusual behavior, e.g., inactivity in the morning. To determine if there is unexpected behavior or if the resident is no longer active, the first step is to try to contact this resident with a mobile application implemented for this purpose. If no response is received after a predefined time, the situation must be escalated, sending a message to a community platform. This platform, where family members and/or care givers are registered, can then initiate the next steps and, in the event of an emergency, notify responsible persons directly.

**Figure 13:**
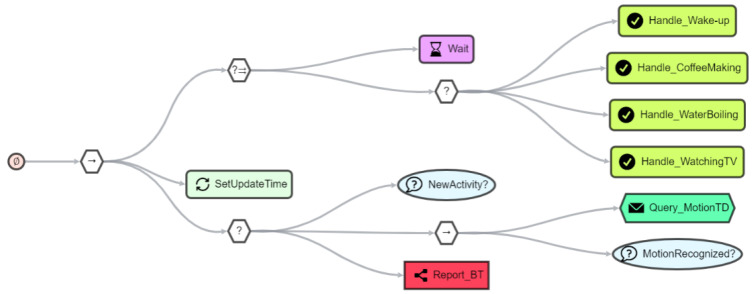
Use-Case emergency checking SBT.

Figure 13 shows an SBT of the *functional agent* we use in our use case to listen for incoming activity events (bright green *handle event node*). While listening, a timer (*wait node*) is set in parallel. If an event is received or the timer has expired, the parallel execution of the sub-BT is successful. In the next steps, the timer is reset (*update node*) and the agent knowledge (*condition node*) as well as the registry (*query node*) are queried about the last activities. If no activity has been received or no motion has been registered by a *monitor agent* within a defined time, further steps must be taken.

**Figure 14:**
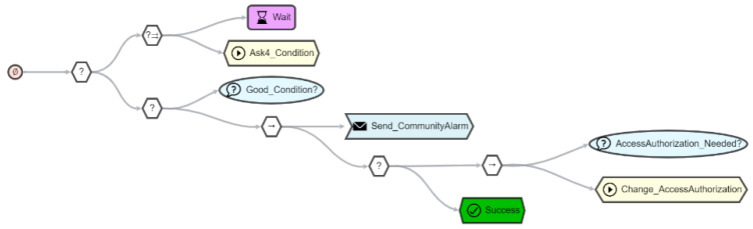
SBT for the user and community platform interaction.

If the next escalation level is reached and the resident is to be contacted directly to inquire about his or her condition, the SBT in Figure 14 is used. To make contact, it must be assumed that the resident will not respond. For this reason, a timer has been defined. Parallel to this timer, a message (*action node*) is sent to the registry, which forwards this message to the mobile application of the resident. The resident then has the opportunity to respond with a predefined description of his or her condition. Finally, the resident’s response is evaluated with a *condition node* and if there is an emergency, an appropriate message is sent to the community platform (via “Send_CommunityAlarm” *plug-in node*). The notified emergency contact can then try to reach the resident by themselves. If no reply is received, the person may ultimately go to the resident’s place and pass the entrance to the home, e.g., via face recognition, which allows him to enter in emergency cases. The agent enables this by using the response from the community platform to grant the specified person access via the face recognition TD available in the registry.

## 9. Related Work

There are several mechanisms to describe things that expose resources and how to interact with them. The Hydra Core Vocabulary [28] is a vocabulary to create hypermedia-driven web APIs. Hydra focuses on the semantic web using HTTP and JSON-LD. While the concept of forms in a TD and operations in Hydra are quite similar, the WoT TD supports a broader range of protocols, such as MQTT and COAP. The Hypermedia Control Ontology and the JSON Schema in RDF vocabulary, as part of the TD, allow the description of legacy devices that do not support semantic payloads in any way. Another mechanism to describe web services is the OpenAPI specification (https://swagger.io/specification/ (accessed on 25 October 2021)). It uses JSON schema to design the data model. OpenAPI does not support the use of semantic annotations; therefore, making it difficult to use a different domain knowledge, such as *iotschema*. Furthermore, OpenAPI only supports HTTP.

The LinkSmart Thing Directory (https://github.com/linksmart/thing-directory (accessed on 25 October 2021)) is an implementation of the TDD, which provides persistent storage and multiple query endpoints for TD metadata. It supports authentication, authorization, and DNS-SD discovery, while offering no SPARQL endpoint to query RDF. Further, it does not support exposing things or mapping a heterogeneous payload of devices. Node-RED (https://nodered.org/ (accessed on 25 October 2021)) is a flow-based tool to interconnect devices and services that support WoT TDs through a plugin (https://github.com/thingweb/node-red-contrib-web-of-things (accessed on 25 October 2021)). The plugin works on the level of the TD information model, ignoring any additional annotated semantics. Therefore, leaving payload mapping and metadata querying up to the developer.

The Topology Orchestration Specification for Cloud Applications (TOSCA) [29] models applications and their dependencies to deploy them across multiple clouds while the Open Cloud Computing Interface (OCCI) [30] focuses on the standardization of a common API for cloud management. Both standards focus on interoperability in the deployment of applications in clouds and not on the interconnection of arbitrary services, as they do not have ways to describe payload schemas or interaction affordances.

Security Assertion Markup Language (SAML) (http://docs.oasis-open.org/security/saml/Post2.0/sstc-saml-tech-overview-2.0.html (accessed on 25 October 2021)) is an open standard for authentication and authorization. Unlike the OAuth 2.0 protocol, SAML is designed for authentication and authorization, while OAuth 2.0 only for the latter. OIDC, as an extension of OAuth 2.0, is used for authentication. While SAML provides encryption for identifiers, attributes, and assertions, OAuth 2.0 and OIDC completely rely mostly on TLS. SAML assumes that the client is a web browser, thus, making it hard to implement mobile applications or applications across multiple security domains. These application types will work out of the box when using OAuth 2.0.

The area of human activity recognition that our use case is part of has been an active field of research for many years now. A review of the studies up until 2019 can be found in [31]. Extensive work has also been done in this field with the focus on non-intrusive load monitoring, not only via appliance detection (see among others [8,9,10]), but also via activity recognition on the energy consumption directly (see [11]).

As for the generation of synthetic data, *TraceGAN* [14] is a solution to generate appliance power using a GAN. It can be used to generate slightly different energy patterns for training NILM applications. The approach generates the power consumption of different appliances by defining one label for each appliance, but only uses one model for all appliances. This seems to be an elegant and efficient solution, but some issues are not solved. It is very hard to generate for example different programs or compositions of a single appliance by just using one label per appliance. Even if the GAN generates different programs or compositions, they are chosen completely randomly. Therefore it is not possible to define which program or which element of a composition should be generated. Moreover, important information, such as the duration of of an appliance usage, cannot be defined with this solution, which results in randomly generated duration of power usage. This does not take the real behavior of an inhabitant into account, who, for example, watches TV for a long time on a weekend, but only sporadically during the week. This lack of taking daily behavior into account is also a problem for the time of day that an appliance, e.g., the washing machine, kettle, or coffee machine is used. Therefore while it is possible to generate data that are technically close to real-world patterns, it is not when it comes to identifying behavioral events, such as wake-up time.

*SynD* [13] also generates data for NILM applications. This solution takes times when a specific appliance is taken into consideration, which can reflect the usual behavior of an inhabitant partially, but does not consider different behavior on different days of the week, or even the temporal dependency of activities that normally follow each other. Therefore, it does not completely model human behavior. For appliances that are controlled by the resident, such as a TV, they use an interpolation policy to mimic different power-on durations. For appliances with programs, such as a dishwasher, *SynD* only uses already recorded data. These power consumption patterns are copied randomly into a specific time slot. This leads to a relatively realistic power consumption output, but only with patterns of the appliances, which an ML service has already seen. This fact can lead to ML models, which overfit to these patterns.

## 10. Discussion and Conclusions

In this paper, we presented an approach to address the evolving cloud-to-thing continuum with a secure IoT registry and an agent-based orchestration framework, which we combined to support semantic orchestration of IoT use cases in federated cloud environments. With our proposed architecture, we have shown that semantics at various levels facilitate the handling of heterogeneous multi-cloud environments for application developers. Using our approaches and components, it is possible to map vendor-specific payload to increase interoperability, and search for functionality across clouds using the thing registry. We integrated security and privacy mechanisms based on well-established industry protocols, such as OIDC, OAuth 2.0, and UMA 2.0, which allow thing owners to authorize access to resources on a very fine-grained level.

We introduced the concept of virtual sensors based on ML models, which allow abstracting raw sensor data and linking the sensor information with semantic descriptions on a logical level. Using virtual sensors, events detected in an environment become available under a new, more abstract description, which can be exploited in scenarios that require information on this more abstract level. An interesting approach for a virtualization layer representing physical sensors is presented in [32]. Our virtual sensors are based on ML models and use signals from physical sensors (which, in principle, can be used in various virtual sensors) and, hence, do not represent them. The term ‘virtual sensor’ was introduced previously in the context of data fusion and aggregation [3]. By conceptualizing ML-based services as sensors, we emphasized that the output of ML models, such as that of sensors, is essentially incomplete and requires, as a complement, further semantic metadata that set the context of the sensor readings.

For implementing virtual sensors, we used artificial neural networks to interpret and enrich the base level sensor information populating the semantic level with machine-readable knowledge. This allowed us to leverage the ability of networks to achieve high accuracies on narrow tasks, while leaving the higher-level reasoning and behavior to more explainable and trustworthy white-box approaches.

The synthetic data generation (see Section 5) enables developers to test their applications or to use pre-trained ML services in new environments. It is possible to simulate a new household and the daily behavior of its inhabitant and, hence, synthesize relatively realistic data to fine-tune the models to the new circumstances, to get a better performance, without waiting months to gather real data.

We illustrated the approach with a use case from an assisted living scenario. The detection of human activity, or lack of it, is particularly important in this domain, specifically in an emergency case, where such information can play a critical role. In the future, almost every household will be equipped with a smart meter. Detecting activities using smart meter data give the potential to deploy and scale the approach. To achieve this, we combined a thing registry, agent-based orchestration, and a power disaggregation approach to detect human activities in a non-intrusive way—that is easy to scale, transfer, and deploy.

In our hybrid approach, classical AI in the form of AJAN, a multi-agent system (MAS), is used at the semantic level. AJAN agents represent, on the one hand, real (but also synthetic) sensors that build up global domain knowledge with the Thing registry. On the other hand, based on this pre-processed information, specialized more complex AJAN agents can be used to respond to critical situations, for example, in the area of ambient assisted living. For this purpose, AJAN uses the provided RDF-based data of the domain knowledge for decision making and communicates directly with the services to be used, using our lifting approach. The behavioral instructions of an agent are thereby available to the user in a human-understandable way. According to [23], web-based MAS have been rather overlooked until today. However, this aspect has changed in recent years. With the advent of WoT, LD, and other W3C standards, voices are getting louder to implement web-based MAS using those [24]. With AJAN, we have a promising approach to respond to these voices.

Obviously, the IoT ecosystem is currently growing at a rapid pace, so ongoing developments will continue to be monitored and taken into account.

## Figures and Tables

**Table 1:** Statistics and results of appliance detection. The window size and activity duration are listed in minutes.

	Washing Machine	Dishwasher	Coffee Machine	Kettle
max power	2050	1850	1600	2950
window size	30	45	15	7
activity duration	134±27	145±38	19±6.2	3.2±1.1
recall	1.0	0.9996	0.88	0.97
precision	0.9993	0.93	0.92	0.72

**Table 2:** Precision and recall of the first stage of wake-up detection.

	Asleep	Recently Awake	Awake
precision	0.861	0.960	0.969
recall	0.978	0.816	0.975

## Data Availability

Not applicable.

## Abbreviations

The following abbreviations are used in this manuscript:

IoT	internet of things
ML	machine learning
IAM	identity and access management
TPC	total power consumption
NILM	non-intrusive load monitoring
WoT	web of things
LD	linked data
RDF	resource description framework
RDFS	RDF-schema
OWL	web ontology language
TD	Thing Description
JSON	javascript object notation
IRI	internationalized resource identifier
AJAN	accessible java agent nucleus
BMWi	Bundesministeriums für Wirtschaft und Energie
BOT	building topology ontology
API	application programming interface
RML	RDF mapping language
XML	full extensible markup language

DFKI	German Research Center for Artificial Intelligence
TDD	Thing Description Directory
TDT	Thing Description Template
OAuth	Open Authorization
OIDC	OpenID Connect
UMA	user-management access
GUI	graphical user interface
